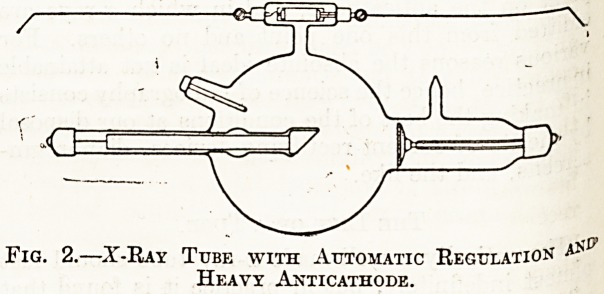# The X-Rays

**Published:** 1912-07-06

**Authors:** Alfred C. Norman

**Affiliations:** House Surgeon at Sunderland and Durham County Eye Infirmary, Sunderland.


### ELECTRICITY IN MODERN MEDICINE.*

### XIV.—The X-Rays.

By ALFRED C. NORMAN, M.D. Edin., House Surgeon at Sunderland and Durham County Eye Infirmary, Sunderland.

### The Modern X-Ray Focus Tube.

To sum up with regard to the appearance of a working *x*-ray tube and the information to be gained therefrom:—

Fluorescence indicates what portions of the walls are being bombarded by *x*-rays or cathode rays; a properly working tube should, therefore, be sharply divided into a light and a dark hemisphere. A tube wrongly connected with the coil shows diffuse patches of fluorescence, but no line of demarcation. A tube, through which some reverse current is passing, even when properly connected, shows irregular patches of fluorescence in the hemisphere that under normal conditions would be dark (see fig. 2, page 248).

An ideal *x*-ray tube, working on an ideal (*i.e.*, an absolutely unidirectional) source of electricity, is one in which the cathode rays arise from the concave electrode only and are brought to a focus at a single point on the anticathode, and in which *x*-rays are emitted from this one point and no others. For various reasons the absolute ideal is not attainable in practice, hence the science of radiography consists in making the best of the conditions at our disposal by means of current rectifying devices, diaphragm-screens, and the like.

### The Life of a Tube.

Theoreticelly a well-made *x*-ray tube should last almost indefinitely, but in practice it is found that after a certain amount of use all tubes lose their efficiency and have to be discarded for new ones. This is due to the fact that the residual gas in a tube tends to become less and less until a stage is reached at which there is, as nearly as possible, an absolute vacuum, and then it becomes impossible for us to force any current through the tube at all. It is generally supposed that this gradual diminution is due to minute particles of gas becoming embedded in the walls of the tube as a result of the tremendous velocity at which they travel when electrified. As its gas diminishes, the resistance of a tube to the passage of electricity increases, and at the same time the *x*-rays become more penetrating. Tubes which are highly exhausted are termed *hard*; those with a lower degree of exhaustion—*medium*; and the lowest which can be used for practical purposes are described as *soft*. In future, in discussing tubes we shall constantly use the term hard, medium, or soft as the case may be, and it should be remembered that these terms have a three-fold significance in that they indicate the degree of exhaustion of the tube, its resistance to the passage of electricity, and, most important of all, the penetrating power of the *x*-rays which it is capable of producing.

Reverse current tends to shorten the life of a tube very considerably, because it tears off minute particles of platinum from the anticathode and these rapidly absorb the available gas; in fact, if the current be completely reversed by connecting the tube wrongly to the coil, the former will in a very few minutes become so hard as to be quite useless.

An *x*-ray tube may also be spoilt by rendering it too soft, so that the *x*-rays which it generates are not sufficiently penetrating to be of any practical use. This is usually the result of passing too much current through the tube when it is new and unseasoned. The makers supply soft tubes unless otherwise ordered, so that the tubes may have as long a life as possible before becoming too hard for use, and it is very easy to pass too much current through a soft tube because its resistance is comparatively low. The actual amount of free gas even in a soft tube (at one-millionth of an atmosphere) is of course infinitesimal, but however well it may be exhausted there must necessarily be a certain amount of dissolved gas in the interstices of the metal parts, cathode and anticathode respectively, and when too much current is allowed to pass through the tube these parts become very hot, with the result that the imprisoned gas is liberated into the tube and markedly reduces its vacuum. In this way a tube may be rendered so soft in a few minutes as to be completely useless.

Another way in which an *x*-ray tube is sometimes ruined is by passing so much current through it that the anticathode becomes red-hot and then melts. This always takes a certain amount of time, and there is really no excuse for such an accident because an observant operator would be warned by a bright spot of incandescence which appears at the focus-point on the anticathode some seconds before the metal actually fuses.

Lastly, a tube may be spoilt by perforating it with an electric spark. This usually happens when it has become so hard that its resistance is considerably greater than would be the case if the space between cathode and anticathode were filled with air. It is a safe rule to keep the conducting wires as far as possible from the body of any tube, but when using a hard tube it is absolutely essential to do so, otherwise a spark will be certain to pass through the wall of the tube rather than traverse the high resistance inside it and the tube will be ruined. Sometimes the puncture lets in just enough air to lower the vacuum of the tube to a degree useless for *x*-ray work and then becomes sealed; in other cases it remains permanently open, converting the tube into a simple spark gap. Generally speaking, the latter is the rule, the discharge through the tube suddenly being converted into a shower of sparks and all fluorescence ceasing, though the writer has seen a tube in which the puncture reduced the vacuum to the Geissler degree and then became sealed, with the result that the tube emitted a beautiful violet fluorescence, but of

* Previous articles appeared on Nov. 11, 25, Dec. 9, 30, Jan. 13, 27, Feb. 17, Mar. 9, 30, April 20, May 4, 25, June 8.

course no $x$-rays. As a rule the puncture is so small as to be almost invisible, and in the case of an expensive tube it is then worth while sending it to the makers to be re-exhausted.

### Regulating Devices.

The early types of tube once they became too hard for use had to be discarded, hence they were made as cheaply as possible. By heating these tubes with a spirit lamp every time they were used and thus driving the occluded gases out of the glass it was possible to regenerate them to a certain extent, but at best their lives were short. It was not long, however, before the ingenuity of the maker furnished us with mechanical regulating devices, by means of which the vacuum of the tube could be reduced at will, and from that time tube-making advanced by leaps and bounds; for, with the prospect of a long life, cost became a secondary consideration and, as a result, tubes of large size with heavy anticathodes (which could be used for instantaneous radiography) came into being.

There are a great number of regulating devices on the market, but the writer has always found "Osmo" regulation to be the most satisfactory, because it is exceedingly simple, easily controlled, and there is no limit to the number of times it can be used. Fig 1 illustrates a tube with "Osmo"

Fig. 1.—*X*-Ray Tube with "Osmo" Regulation.

regulation. At O there is a small tubular prolongation into which is sealed a tiny tube of palladium having its external end closed and its other end open and projecting into the lumen of the $x$-ray tube. About an inch and a half of the palladium tube extends outside of the glass and is protected by a small glass thimble. When it is desired to make the $x$-ray tube softer—*i.e.*, to reduce its vacuum—it is only necessary to remove the glass thimble and to heat the tip of the palladium tube to a dull red by means of a spirit lamp. Palladium, when hot, possesses the property of transmitting small quantities of hydrogen, and this, entering the $x$-ray tube, produces the desired effect. It must be remembered that an infinitesimal amount of gas will have an enormous effect on the working condition of the tube, for we are dealing with a tube which contained a hardly appreciable amount to begin with, since it was exhausted to the one-millionth of an atmosphere or less. The palladium should be heated for about three seconds only, and *must be allowed to cool* before the current is turned on; if the reduction is not sufficient the process may be repeated, but great care must be exercised not to lower the vacuum too much. Other precautions to be observed are: not to raise the palladium to a white heat or it may melt and puncture the tube, and not to heat the metal near the glass or the latter may crack.

Other types of regulation may be classified as "automatic." Fig. 2 illustrates a tube with automatic regulation. At the top of the bulb there is a subsidiary bulb communicating with the main tube by a narrow neck. Inside this bulb there is some substance containing gas, and the latter can be liberated by passing an electric current through the bulb. Into the ends of the subsidiary bulb are fused two wires so arranged that when the tube gets hard a spark will pass from the anode to the left-hand wire, the current traversing the bulb and passing through the right-hand wire to the cathode. The current in traversing the bulb liberates gas and, theoretically, the wires can be set so as to keep the tube at a constant vacuum, but in practice this is generally not attained. As a rule it is better to keep the wires as far as possible from the terminals of the tube and to pass a little current through them when necessary to lower the vacuum. All automatic regulators become exhausted in time.

In practice it is advisable never to use any type of regulator when it can possibly be avoided, because after a time the quality of the $x$-rays deteriorates as a result of constant tampering with the vacuum. For radiographing different parts of the body, tubes

Fig. 2.—*X*-Ray Tube with Automatic Regulation and Heavy Anticathode.

of different hardness are required, and at least three degrees should be always at hand. It is most economical to buy soft tubes because they are obliged to become harder in time, and in this way we can get more out of them before the regulator has to be used. Of course, in purchasing a new $x$-ray outfit one hard tube should be obtained, but after this only soft ones need be ordered, for they will grow hard too quickly in any case.

There is a method, however, by which it is possible to keep tubes at an almost constant degree of vacuum if the worker has the sole use of a set and cares to take the trouble. We have seen that too much current softens the tube by generating heat in the metal parts and liberating gases, and that considerably less current hardens the tube by driving the gases into the glass walls; obviously, therefore, there must be a certain current which will just balance these two processes and keep the tube constant. In practice this is found to be the case, and in the next section a simple method of determining and regulating this current for any tube will be described. The writer is in the fortunate position of having sole charge of the $x$-ray room in which he works, and he has often by this method been able to keep tubes in a constant condition for more than a year without once using the regulators with which they are provided.

(*To be continued.*)

## Figures and Tables

**Fig. 1. f1:**
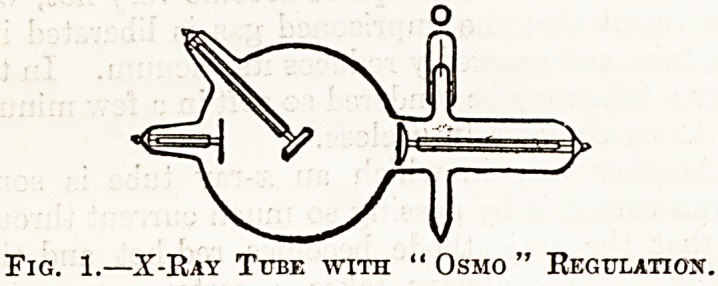


**Fig. 2. f2:**